# Optimization of Ultrasonic-Enzyme Synergistic Extraction of Proanthocyanidins from Jujube: Purification, Characterization, and Bioactivity Study

**DOI:** 10.3390/molecules30030619

**Published:** 2025-01-31

**Authors:** Qiaoshuang Lu, Zheng Ye, Chun Yang

**Affiliations:** 1 College of Food Science and Engineering, Shanxi Agricultural University, Jinzhong 030801, China; z20223035@stu.sxau.edu.cn; 2 Shanxi Institute for Functional Food, Shanxi Agricultural University, Taiyuan 030001, China

**Keywords:** jujube, proanthocyanidin, resin, oxidation, hypoglycemic

## Abstract

Proanthocyanidins have received extensive attention due to their high functional value, but their sources are limited. Therefore, this experiment studied the preparation, biological activities, and characterization of proanthocyanidins from Chinese jujube (*Ziziphus jujuba* Mill. cv. *Muzao*) at different periods, aiming to explore a new source of proanthocyanidins and enhance their utilization value. Through ultrasonic-assisted enzymatic extraction, the optimal extraction conditions for PC from *Muzao* were determined, yielding a proanthocyanidin content of 2.01%. Purification using AB-8 macroporous resin increased the proanthocyanidin content by 11 times. The bioactivity results indicated that proanthocyanidins demonstrated significant in vitro antioxidant activity (scavenging rate ≥ 83.4%) and blood glucose-lowering activity (inhibition rate ≥ 84.7%). Both activities decreased with maturity, while the degree of polymerization also exhibited a positive effect. Mass spectrometry identified a total of 102 compounds, with cyanidin-based compounds being the most abundant, comprising 28 species. The comprehensive research results indicate that the oligomeric proanthocyanidins extracted, purified, and isolated from *Muzao* during the young fruit stage exhibit diverse biological activities and are abundant in content. They can be utilized for the extraction and purification of proanthocyanidins, offering a reference for the expansion of natural sources of proanthocyanidins and the development of functional foods.

## 1. Introduction

Proanthocyanidins (PCs) are polyphenols synthesized as oligomers or polymers of the flavan-3-ol unit through the flavonoid pathway in plants, which have various physiological effects such as anti-oxidation, anti-cancer, anti-obesity, neuroprotective, anti-radiation, and anti-virus [1,2,3]. PCs are widely found in fruits, berries, nuts, and seeds [4], and the most extensively studied source is grapes [5]. In addition, there are also rich amounts of anthocyanins in fruits and vegetables such as apples, waxberries, cherries, and lychees [6,7]. For example, Bindon et al. [8] mentioned that procyanidins are actually the most abundant polyphenols in apples, with a content ranging from 69% to 82% of the total polyphenols in fresh apples; Nemes et al. [9] successfully developed a new process for extracting the unextractable procyanidins from Hungarian sour cherries that are bound to membranes, proteins, and fibers, with an estimated 100–300 mg of PCs in 100 g of fresh fruit. In recent years, proanthocyanidins have attracted extensive attention due to their various active effects, while there is little research on other plant sources, such as jujube, especially *Muzao*, one of the ten Chinese jujube varieties mainly produced in Shanxi Province of China, which has not been studied yet.

*Muzao* has thin skin, a crispy texture, and delicious pulp; it is rich in nutrients and has been used in food therapy for thousands of years. It is not only a good source of nutrition but also contains various bioactive substances, such as vitamin C, polysaccharides, phenols, flavonoids, triterpenes, and saponins [10], which are basic chemical compounds involved in disease management and provide health benefits. PCs are one of the most important secondary metabolites belonging to polyphenols and are strongly related to antioxidant activity. Compared with other edible fruits mentioned above, jujube is known as the “fruit of life” and is widely used as a medicine to treat allergies, constipation, urinary system problems, depression, chronic bronchitis, insomnia, and liver disease in Asian countries, especially in China [11]. Multiple studies have shown that jujube has a stronger antioxidant capacity. Among 62 common fruits, jujube has the highest phenol content (585.52 ± 18.59 mg GAE/100 g) and antioxidant activity (8.33 ± 0.12 μmol Trolox/g) [12]. The free radical scavenging ability of jujube fruit is 2–3 times that of common fruits such as lemon, lychee, apple, avocado, cantaloupe, banana, durian, longan, and papaya [13].

During the continuous development of dates, their nutrient composition changes, and differences in the range of PCs are most likely due to their stage of maturation. Understanding the changes in physicochemical properties and biological activities of jujube during its development is of great significance for the development of its functional active ingredients and functional food. Huang et al. [14] used label-free quantitative proteomics to study the changes in the proteome of jujube at four growth stages. The results showed that the physiological traits of seedless jujube fruit changed significantly during the four growth stages and were significantly correlated with changes in fruit quality. Reche et al. [15] found that the developmental stage of Spanish dates had a significant effect on the physicochemical and antioxidant activity of five date varieties, which can be used as natural antioxidant extracts and proved to be a rich source of phenolic compounds and antioxidants. Hu et al. [16] studied the physicochemical properties and antioxidant activity of Jade Bell Jujube at seven stages of ripening and found that the antioxidant properties gradually decreased as ripening progressed; this study also showed that total sugar and total acid levels increased during ripening, while water, soluble protein, and sugar to acid ratios decreased. These studies proved the importance of studying various physicochemical properties, active ingredients, biological activities, and the structural characterization of jujube during its development and provided references for quality improvement, resource utilization, and functional food development. There have been relevant reports on the study of PCs in jujube [17]. However, due to differences in varieties and development degree, research on the changes in bioactivity and component analysis and identification of purified proanthocyanidins during the development of *Muzao* has not yet been reported. Clearly, it is necessary to conduct research on this issue.

This study aims to explore the bioactivity, composition, and structure of proanthocyanidins in *Muzao* to contribute to the expansion of natural plant sources of PCs. Therefore, this experiment was conducted to determine the optimal process of ultrasonic combined with enzyme extraction of *Muzao* Proanthocyanidins (MPCs) by using the extraction rate of proanthocyanidins as the index of investigation and by using one-way and response surface methods. The MPC extract was purified by using AB-8 macroporous resin, so as to obtain the optimal conditions for the purification and to prepare the purified material. On this basis, the bioactivities of the extracts were compared with those of the purified products; furthermore, the bioactivities and structural and morphological characteristics of the purified and isolated oligomeric proanthocyanidins (OPCs) and polymeric proanthocyanidins (PPCs) were comprehensively evaluated, and finally, OPC was identified in a more comprehensive way. In conclusion, this study is dedicated to fully utilizing the high-performance resources and processing value of *Muzao* as well as providing a theoretical basis for the discovery of natural active ingredients and their development into natural effective antioxidants and functional foods.

## 2. Results

### 2.1. Single Factor Test Results

#### 2.1.1. Effects of Ethanol Concentration and Solid-to-Liquid Ratio

**Figure 1:**
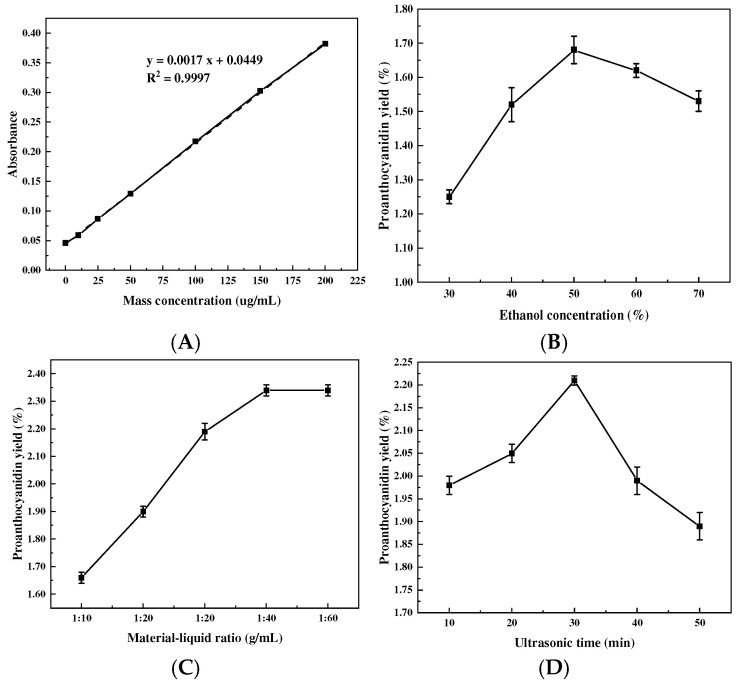

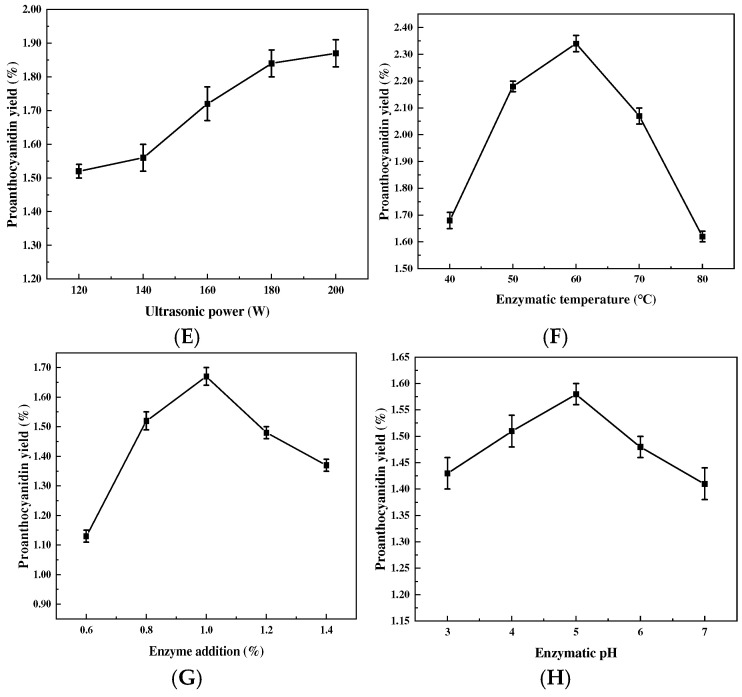
Standard curve of proanthocyanidins (**A**), the effects of ethanol concentration (**B**), solid/liquid ratio (**C**), ultrasonic time (**D**), ultrasonic power (**E**), enzymatic temperature (**F**), enzyme addition (**G**), and enzyme pH (**H**) on the extraction yield of *Muzao*. The values are expressed as means ± SD (*n* = 3).

The results of the one-way test for MPC extraction by ultrasound combined with the enzymatic method are shown in Figure 1. Ethanol is often used for proanthocyanidin extraction due to its advantages of low cost and toxicity, and in this study, food-grade ethanol was configured as a solvent in aqueous ethanol solutions at different concentrations in order to minimize the potential toxic effects. Figure 1B shows that within a certain range, the production of MPCs increases with the increase in ethanol percentage, and the yield of MPCs reaches its highest level when the ethanol concentration is 50%, which may also be due to the fact that heating and sonication treatments significantly shortened the extraction time and ethanol aqueous solution requirement [5]. From Figure 1C, it can be seen that as the solid-to-liquid ratio increases, the contact area between the material and the solvent enlarges, leading to a gradual increase in the extraction rate of MPCs. However, when the solid-to-liquid ratio increases from 1:40 to 1:60, there is little change. This is due to the incomplete dissolution of PCs when the extraction solvent ratio is low. However, when the extraction solvent exceeded a certain ratio, the resistance to ultrasonic cell breakage increased, resulting in a decrease in the yield of PCs [18]. For economic considerations, the material/liquid ratio was chosen to be 1:40.

#### 2.1.2. Effects of Ultrasound Time, Power, and Temperature

From Figure 1D, the yield of proanthocyanidins showed a trend of increasing and then decreasing with increasing ultrasound time, and when the ultrasound time exceeded 30 min, the yield of proanthocyanidins instead began to decrease, which was attributed to the fact that excessive prolongation of the ultrasound time triggered the oxidation or degradation of proanthocyanidins [19]. Sun et al. [20] used enzymatic hydrolysis and ultrasonic extraction sequentially, which required a total of 70 min, whereas in this study, the combination of ultrasound and enzymatic methods significantly shortened the extraction time. The effects of ultrasound power and temperature show a similar trend (Figure 1E,F), where an increase in ultrasound power promotes an increase in temperature, which in turn, promotes the degradation of OPCs [21]. Thus, when both are increased, it promotes MPC extraction and then decreases MPC extraction after reaching a plateau.

#### 2.1.3. Effects of Enzyme Addition and pH

The use of enzymes can optimize the release of bioactive compounds from plant cells through cell wall disruption and extraction [22]. As can be seen from Figure 1G, the yield of proanthocyanidins shows a trend of first increasing and then decreasing with the addition of cellulase, and the yield of proanthocyanidins declines when the addition exceeds 1%. This may be because, at low levels of cellulase, all enzyme active sites bind to the substrate until saturation is reached. Further increases in enzyme quantity may lead to increased competition between the substrate and the enzyme, inhibiting the exposure of the enzyme’s active centers, thereby reducing mass transfer between enzyme particles and the substrate and causing unfavorable interactions [20]. In addition to enzyme quantity and temperature, the most important factor affecting enzymatic hydrolysis is the acid-base environment of the reaction. Proanthocyanidins are more stable in acidic environments compared to alkaline environments. Therefore, this experiment investigated the yield of MPCs within the pH range of 3 to 7, and it was found that the highest yield of MPCs was obtained at pH 5 (Figure 1H). This may be because proteins are one of the main impurities in the extract, and changes in pH can alter the net charge of proteins, tending to change the electrostatic interactions between proteins and membranes as well as between proteins themselves [22].

The experimental results showed that the optimum conditions for each single factor were an ultrasonic power of 200 W, a solid/liquid ratio of 1:40, an ethanol concentration of 50%, an ultrasonication time of 30 min, an enzymatic temperature of 60 °C, a cellulase addition of 1%, and an enzymatic pH of 5. Similar optimum process conditions for the extraction of proanthocyanidins were also observed in many studies [5,20,23,24].

### 2.2. Response Surface Method (RSM)

#### 2.2.1. Establishment of the Regression Model

The main advantage of the RSM is that it reduces the number of experimental trials required to estimate multiple factors and their interactions [25]. Based on single-factor experiments, Box Behnken was used to conduct a three-factor three-level central combination experiment design on the three influencing factors of extraction temperature (A), solid/liquid ratio (B), and enzyme addition amount (C). A total of 17 sets of experiments were designed, including 5 sets as central point repeated experiments [26]. The experimental results are shown in Table S1 (Supplementary Materials). The data in Table 1 were fitted to obtain a quadratic multinomial regression equation:

Y = 2.004 + 0.1375 A + 0.0050 B + 0.0175 C + 0.0675 AB + 0.0025 AC + 0.1475 BC − 0.19325 A^2^ − 0.26825 B^2^ − 0.06825 C^2^ (1)

#### 2.2.2. Regression Equation and Variance Analysis

The applicability of the model usually depends on the realization of significant regression coefficients (R^2^) and misfit insignificance, and checking the misfit term of the RSM model is essential because the inability of the data to fit the model will lead to inaccurate or misleading predictions [27]. As can be seen from Table S1, in the regression model, *p* < 0.01 indicates that the difference between the quadratic regression equation models is highly significant. The correlation coefficient of the model, R^2^ = 0.9202, and the corrected coefficient of determination, R^2^Adj = 0.8175, indicating that the actual values of the model fit well with the predicted values, and the misfit term, *p* = 3.07 > 0.05, indicate that the misfit is not significant and the test error is small. Therefore, the model can be used for the analysis and detection of MPC yield.

#### 2.2.3. Interaction Analysis of the Response Surface

**Figure 2:**
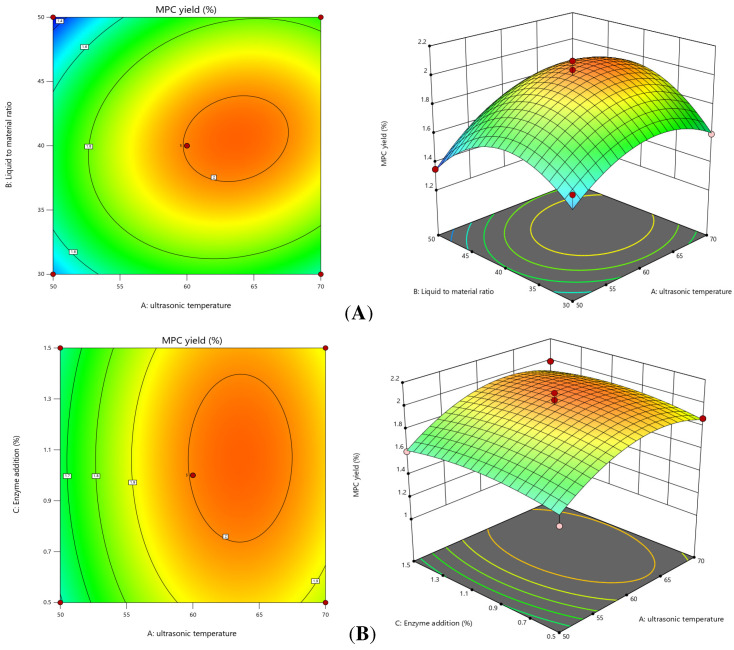

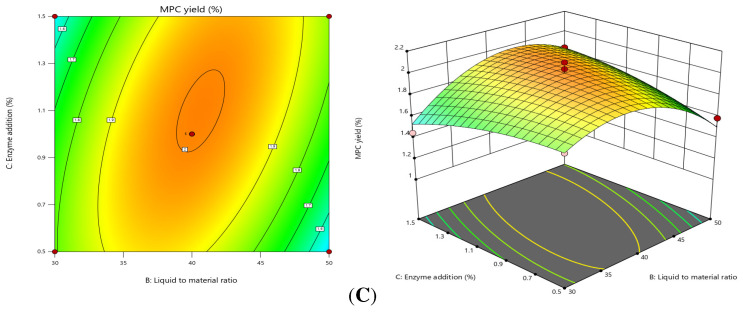
Response surface plots for interactions between various factors on the yield of MPCs. Extraction temperature and liquid ratio (**A**), extraction temperature and enzyme addition (**B**), and liquid ratio and enzyme addition (**C**).

According to the regression model, fixing any two levels at the zero level, the response surface plots and corresponding contour plots of the interaction of the other two factors can be obtained, reflecting the effect of the interaction of the factors on the extraction rate. The interactions of the factors in this experiment are shown in Figure 2, and the interaction between temperature and the material/liquid ratio is significant, and its contour line is elliptical. The interaction between temperature and the material/liquid ratio is significant, and the contour line is elliptical. Comparing the graphs, it can be seen that the temperature and the material/liquid ratio have a greater effect on the extraction rate of date anthocyanins, which is reflected in the steeper surface of Figure 2A,B. The effect of enzyme addition on the yield of MPCs was not obvious, as shown in the relatively flat surface of Figure 2C.

#### 2.2.4. Verification Test

The design-expert 13 software calculated the best parameters for the extraction: the extraction temperature of 63.81 °C, the liquid ratio of 1:41.35, and the enzyme addition amount of 1.14%. In this process, the extraction rate can reach 2.03% theoretically. According to the experiment of the above process parameters, the enzymatic temperature, solid/liquid ratio, and enzyme addition are 64 °C, 1:41, and 1.14%. Under this process condition, the actual measurement of the average rate is 2.01%. Compared with the theoretical prediction, the relative error is about 0.02%, and repeatability is very excellent, indicating that the model can better predict the relationship between MPCs and the experimental conditions and has a certain practical value, and this condition and the yield were better than in the study by Tian et al. [28].

### 2.3. Adsorption and Desorption of AB-8 Macroporous Resin

#### 2.3.1. Effect of Sample Concentration and Adsorption Time on the Adsorption Rate

**Figure 3:**
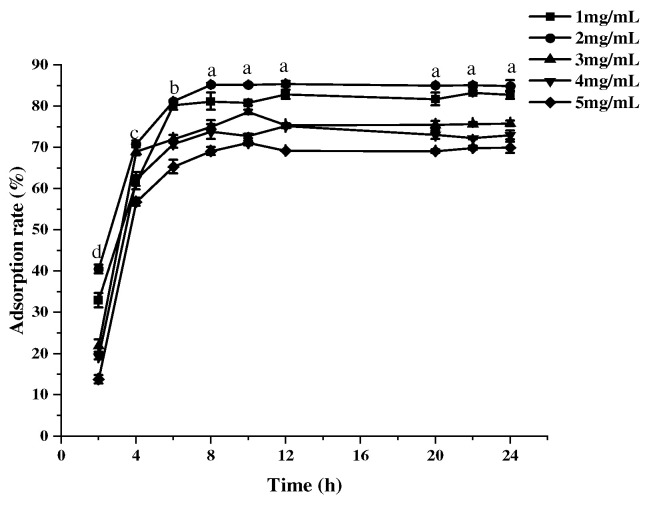
The effect of different sample concentrations on resin adsorption rate with adsorption time. The values are expressed as means ± SD (*n* = 3). Lowercase letters indicate significant differences in the adsorption rate at different times.

Macroporous resin is a styrene-type non-polar copolymer, used as an adsorbent to remove colloid, starch, sugar, fat, tannins, and inorganic salts in the preparation of crude extract [29]. As the concentration of the MPC sample solution increased, the adsorption rate showed a tendency to increase and then decrease, reaching a maximum at a concentration of 2.0 mg/mL (Figure 3). This is because in a certain concentration range, the adsorption capacity of the resin increases with increasing concentration, but after reaching adsorption saturation and equilibrium [30], the adsorption capacity of the resin no longer increases, and the adsorption rate decreases when the concentration continues to increase. In addition, the adsorption rate of AB-8 macroporous resin on MPCs increased with the increase in adsorption time, and the adsorption rate basically did not change anymore after 8 h of adsorption and reached the adsorption equilibrium, which indicated that the kinetic characteristics of the resin AB-8 were obvious [31], which was suitable for the adsorption study of MPCs, and that the adsorption time of the static adsorption test should be more than 8 h.

#### 2.3.2. Effect of the Loading Flow Rate on the Adsorption Rate

**Figure 4:**
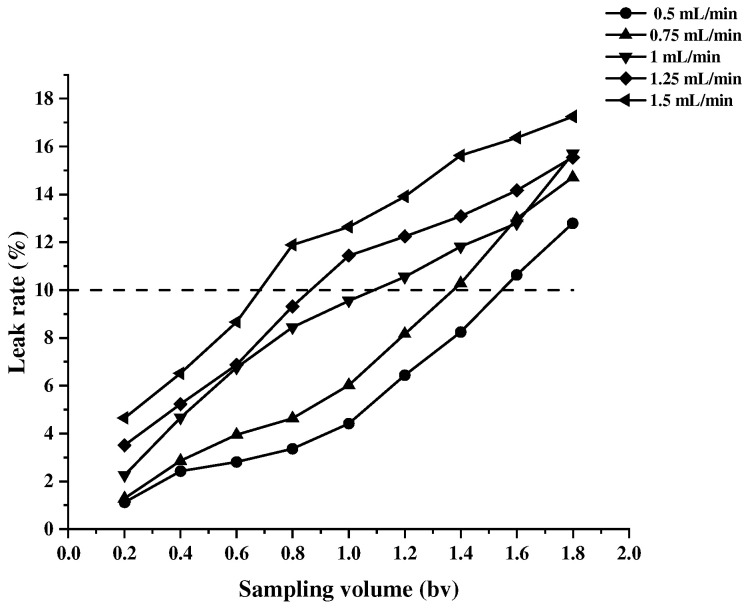
The effect of the loading flow rate on the resolution rate of MPC. The dashed line indicates that the adsorption resin is approaching or has reached its saturation adsorption capacity.

We completed wet loading the pre-treated macroporous resin onto the column, measuring the concentration of proanthocyanins in the effluent adsorption residue, and analyzing the effect of the loading flow rate on the adsorption efficiency of AB-8 resin. As the volume of the effluent increases, the faster the flow rate, the higher the concentration of adsorbed residual liquid (Figure 4). When the flow rate is 1.25 mL/min and 1.5 mL/min, the concentration of the adsorbed residual liquid exceeds the leakage point concentration when the volume of the effluent is 0.8 bv, whereas the concentration saturated by the resin adsorption is reached when the flow rate is 0.75 mL/min and 0.5 mL/min, and the volume of the effluent is about 1.5 bv. This is because, when the sampling flow rate is too fast, the residence time of the proanthocyanidin sample solution in the chromatographic column is too short, the contact with the resin is not sufficient [32], and the resin does not have time to adsorb the proanthocyanidin into the internal pore size of the resin, so most of the proanthocyanidin flows out with the solution, which results in the wastage of the sample solution. When the flow rate is 1 mL/min and 0.75 mL/min, the adsorption capacity of the resin for the proanthocyanidin sample solution is relatively similar, so choosing 1 mL/min as the sample flow rate can save time and instrument use costs.

#### 2.3.3. Effect of Loading Volume and Sample pH on the Adsorption Rate

**Figure 5:**
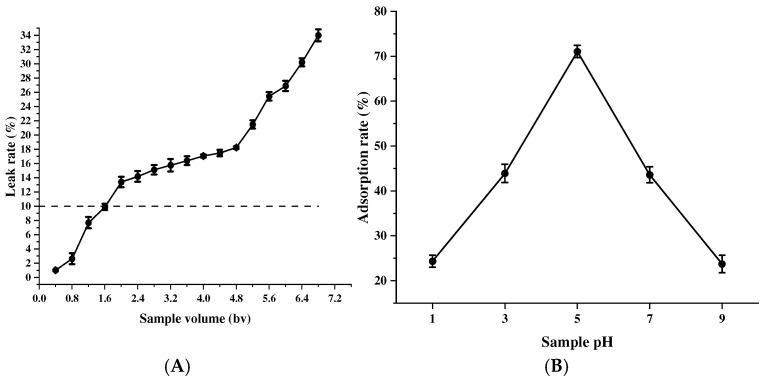
Effect of the loading volume (**A**) and sample pH (**B**) on the adsorption effect of MPC. The values are expressed as means ± SD (*n* = 3). The dashed line indicates that the adsorption resin is approaching or has reached its saturation adsorption capacity.

In the dynamic adsorption process, the adsorption volume will increase with the increase in the sample volume, and too large a sample volume will lead to the waste of proanthocyanidins in the sample solution as well as the difficulty of resin regeneration, but too small a sample volume will reduce the efficiency of the experiment, so the optimum sample size (i.e., the leak point concentration) is usually the volume at which the adsorbed residue reaches 10% of the sample concentration [33]. At a sample volume of about 1.6 bv, the concentration of the adsorbed residue reached the leak point concentration (Figure 5A), so a sample volume of 1.6 bv was selected. The adsorption rate of resin AB-8 on MPCs showed a tendency to increase and then decrease with the increase in pH (Figure 5B), and the adsorption rate under acidic conditions was obviously higher than that under alkaline conditions and reached the maximum value at pH = 5.0. This may be due to the fact that proanthocyanidins with multiple hydroxyl groups are weakly acidic [34], and the adsorption effect is better under acidic conditions, so the adsorption process should be carried out under acidic conditions, and the pH value should be around 5.0.

#### 2.3.4. Effect of Different Elution Conditions on the Desorption Effects

**Figure 6:**
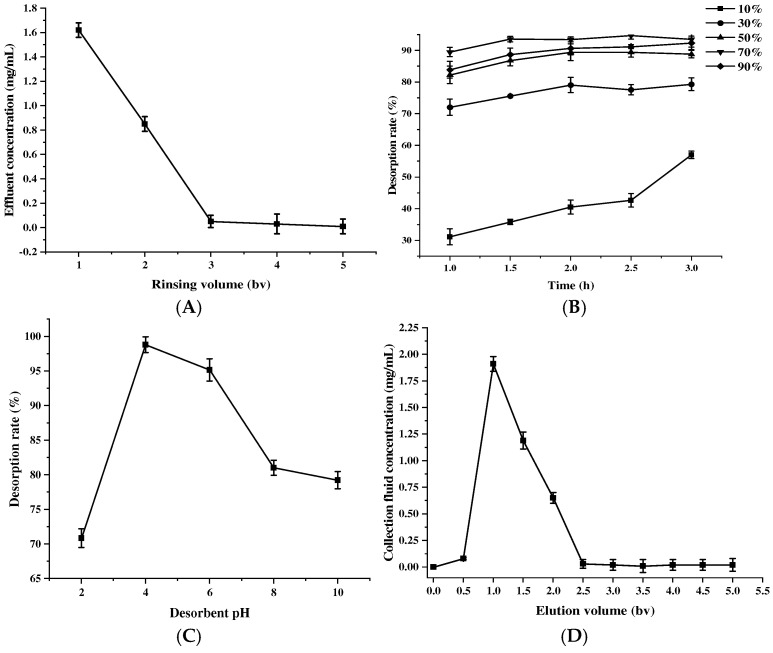
Effect of the rinsing volume (**A**), desorption time and eluent concentration (**B**), desorbent pH (**C**), and elution volume (**D**) on the desorption effect of proanthocyanidins from *Muzao*. The values are expressed as means ± SD (*n* = 3).

The drenching process makes the proanthocyanidins that are not adsorbed be quickly rinsed off by the drenching liquid, and by continuing to increase the drenching volume, the MPCs will not be rinsed off, but by increasing the rinsing volume of distilled water, more impurities such as large molecular proteins and polysaccharides can be eluted, and the purity of the product can be improved. The optimal rinsing volume was determined to be 3 bv (Figure 6A), taking into account the cost, time, and waste of excessive distilled water.

Desorption capacity varies significantly with the proportion of ethanol in water [31]. With the increase in desorption time, the desorption first increased and then smoothed out, and the maximum desorption rate was reached at about 2 h, and the maximum desorption rate was achieved when the concentration of 70% ethanol was desorbed (Figure 6B). This may be because proanthocyanidins are adsorbed on the resin due to hydrogen bonding and the low volume concentration of the eluent is less polar than the force between the macroporous resin and the sample solution [35], and solvent mixtures with higher polarity improve the extraction of proanthocyanidins and break the bonds between the phenolic compounds and the substrate [36].

Moreover, with the increase in eluent pH, the desorption reached the best value at pH = 4 (Figure 6C). This is because PC is stable under weakly acidic conditions, due to the higher stability of A-type C-O-C bonds compared to B-type C-C single bonds, which reduces the degradation rate. Strongly acidic conditions lead to depolymerization and tautomerization, while alkaline conditions promote oxidation reactions, both of which accelerate PC degradation [37]. The concentration of MPCs is characterized by an initial increase followed by a decrease, as shown in Figure 6D. When the eluent dosage is less than 0.5 bv, the sample concentration in the effluent tends to be zero, and the effluent at this time may be water or other impurities. When the eluent dosage was 1.0 bv, the sample concentration in the effluent was the highest at 1.87 mg/mL; when the dosage was greater than 2.5 bv, the sample concentration in the effluent tended to be zero, with less residue. Therefore, 2.5 bv is the optimal eluent dosage for the desorption of saturated AB-8 resin.

### 2.4. Antioxidant In Vitro

#### 2.4.1. Effects of Different Maturity on Antioxidant Activity

**Figure 7:**
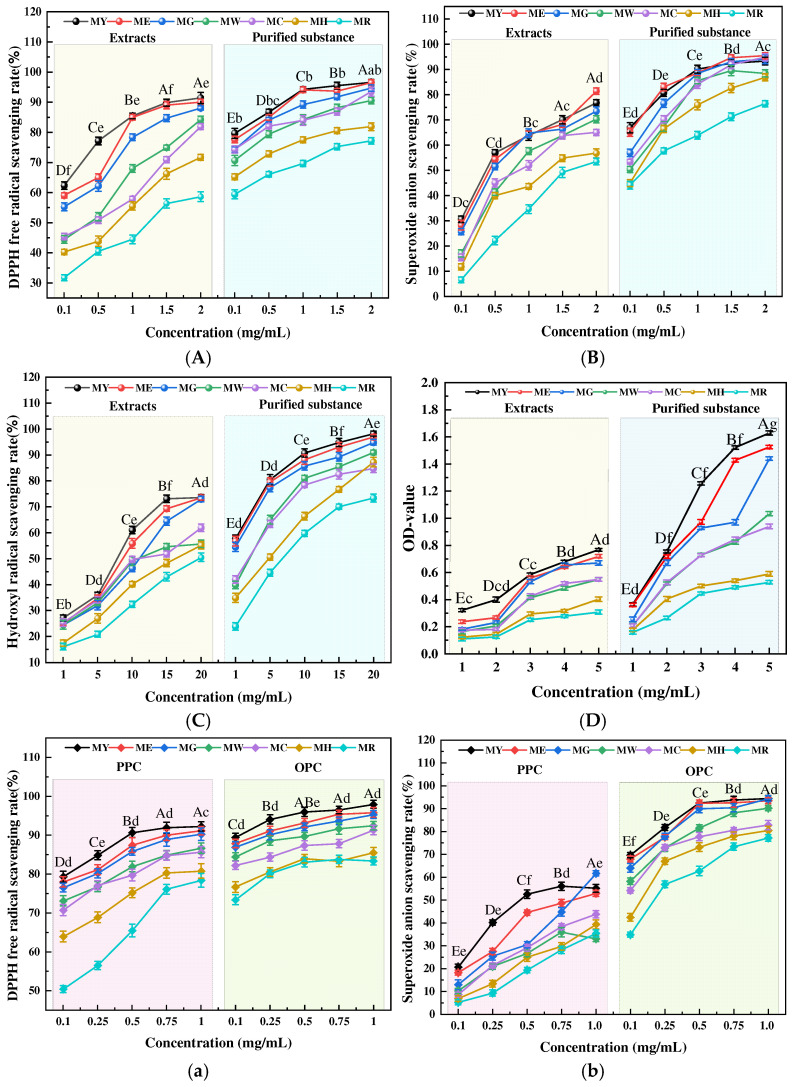

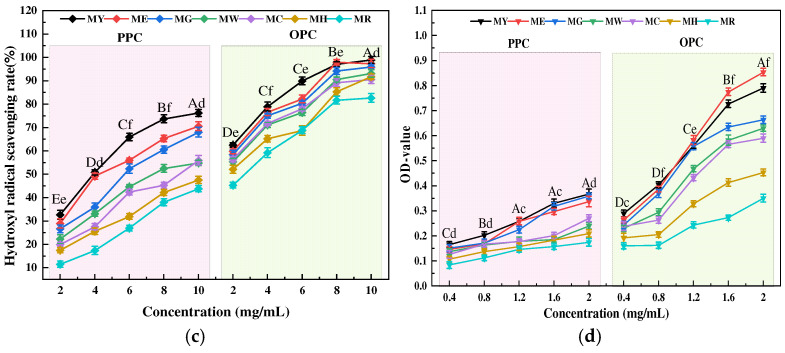
Various antioxidant activities of DPPH radical scavenging ability (**A**), superoxide anion scavenging rate (**B**), hydroxyl radical scavenging rate (**C**), and the reducing power (**D**) between crude extract and purification; various antioxidant activities of DPPH radical scavenging ability (**a**), superoxide anion scavenging rate (**b**), hydroxyl radical scavenging rate (**c**), and the reducing power (**d**) between PPC and OPC. MY, ME, MG, MW, MC, MH, and MR represent different periods of *Muzao*. The values are expressed as means ± SD (*n* = 3). Different letters indicate significant differences (*p* < 0.05). Uppercase letters indicate significant differences in antioxidant activity among different concentrations; lowercase letters indicate significant differences in antioxidant activity among different periods.

As shown in Figure 7, the antioxidant capacity of proanthocyanidins decreased with the maturity of *Muzao* and continued to increase to a stable level with the increase in concentration. At the same concentration, the antioxidant activities of the fractions obtained from the extraction, purification, and isolation processes of *Muzao* of different maturity levels were significantly different (*p* < 0.05), among which the difference was greatest at MY and MR. Taking extracts as an example, the highest DPPH free radical scavenging rate, superoxide anion scavenging rate, hydroxyl free radical scavenging rate, and reducing power were 85%, 63.8%, 73.1%, and 0.77, respectively, at MY, but decreased to 44.5%, 22.2%, 43%, and 0.31 at MR.

On the one hand, this may be due to the continuous decline in the content of various nutrients and active components during the development stages of *Muzao*. On the other hand, it has been reported that the mature stage of plants exhibits an effect on the antioxidant activity of PCs. Katherine et al. [38] found that Ocimum basilicum L. maturity significantly affected the total anthocyanin content (2.07–9.72 mg/g DW) and the concentration of the four most abundant anthocyanins; plant maturity also had a significant effect on the measured FRAP (ferric-reducing antioxidant power), and the reducing capacity (3.50–28.73 mmol/100 g DW) also had a significant effect. Wang et al. [39] also found that the diversity of ripening stages of fire buckthorn fruits resulted in proanthocyanin antioxidant activity differences. The results showed that the antioxidant activity of MPCs was significantly affected by maturity.

#### 2.4.2. Effect of Resin Purification on Antioxidant Activity

Figure 7A–D shows the comparison of antioxidant activity between the extract and the purified proanthocyanidin of *Muzao*. Among them, in the concentration range of 0.1–2 mg/mL, the scavenging rate of DPPH free radical was in the order of purification (59.4–96.6%) > extract (31.7–91.4%). The highest inhibition rate was similar to that of Lycium barbarum (97.76%) [40] and grape seed (93.30%) [41] but higher than that of jujube (70%) [42] and Rhizome of Fagopyrum debtors (72.11±0.42%) [43], grape seed (81.10%) [20], and raspberry (92%) [44]. In the concentration range of 0.1–2 mg/mL, the superoxide anion clearance was ranked as purified (44.1–93.3%) > extract (6.6–76.8%). In the concentration range of 1–20 mg/mL, the hydroxyl radical scavenging rate of extracts (16.1–73.6%) was lower than that of purified extracts (23.9–98.2%), higher than Shi et al.’s [44] study on raspberry: 0.1 mg/mL cleared more than 90% of hydroxyl free radicals and was stronger than vitamin C (57%). In the concentration range of 1–5 mg/mL, the maximum reducing power of the purified substance was 1.63, while the maximum reducing power of the extract was only 0.77. Additionally, the highest superoxide anion scavenging rates in chokeberry [45] and sour jujube seed [46] reached 90% and 65%, respectively, with reducing powers of 0.6 and 1.2. The antioxidant capacity of proanthocyanidins in these two fruits is significantly lower than in this study. Meanwhile, purification using AB-8 macroporous resin significantly reduces the proportion of impurities, thereby enhancing the ferric-reducing antioxidant power (FRAP) and DPPH· scavenging activity [47]. It can be seen that the antioxidant capacity of the purified substance after purification by macroporous resin is significantly higher than that of the extract.

#### 2.4.3. Effect of Different Polymerization Degrees on Antioxidant Activity

As shown in Figure 7a–d, to clarify the effects of different polymerization degrees of proanthocyanidins in purified products on the antioxidant activities of MPC, the DPPH method, salicylic acid method, o-Benzenetriol method, and iron ion reduction method were used to detect the antioxidant activities of PPC and OPC after extraction and separation, to compare their antioxidant mechanisms. DPPH radical is used to determine the hydrogen atom or electron donor capacity of a compound [48]. Figure 7a shows that proanthocyanidins with different degrees of polymerization have obvious DPPH radical scavenging capacity in a concentration-dependent way. OPC (73.4–97.9%) > PPC (50.4–92.3%) in the concentration range of 0.1–1 mg/mL. Superoxide anions are precursors of active free radicals and play a key role in the formation of various reactive oxygen species [49]. Figure 7b shows that the results of superoxide anion detection have the same trend as those of DPPH detection. In the concentration range of 0.1–1 mg/mL, OPC (34.8–94.5%) > PPC (5.2–55.1%). Among reactive oxygen species, hydroxyl radical •OH is the most active and dangerous, capable of damaging cellular systems such as DNA, proteins, and lipids [50]. Figure 7c shows that OPC (45.3–98.9%) > PPC (11.5–76.2%) in the concentration range of 2–10 mg/mL. With the increase in the concentration of proanthocyanidins, the inhibitory effect of proanthocyanidins on hydroxyl radicals was gradually enhanced, and the maximum was about 98%.

The higher absorbance of the reaction mixture indicates a higher reducing power [51]. The reducing power of OPC is much stronger than that of PPC, as can be seen in Figure 7d; OPC (0.16–0.79) > PPC (0.08–0.37) in the concentration range of 0.4–2 mg/mL. Klavins et al. [52] found that OPC has the strongest scavenging activity against DPPH, and proanthocyanidins play a major role in antioxidants as part of the total polyphenols. This is similar to the findings of Arimboor et al. [53]: proanthocyanidin content was positively correlated with free radical scavenging activity, and proanthocyanidins containing high amounts of phenolic hydroxyl groups had higher antioxidant activity. Taking the above results and analysis together, we can learn that OPC is the main contributor to the antioxidant activity of *Muzao* after isolation and purification. Additionally, clinical and experimental evidence shows that REDOX imbalance (OS) exists in a variety of acute/chronic diseases, including cardiovascular diseases, neurodegenerative diseases, metabolic diseases, and inflammatory diseases [54]. PC stabilizes and inactivates free radicals by providing an electron to the radical -OH group attached to the phenol ring, terminating the oxidative chain reaction, thereby destroying direct OS damage and the REDOX chain reaction [55], preventing OS-induced DNA damage and promoting DNA repair [56]. Due to its strong antioxidant properties, PC may be a powerful clinical tool for managing these diseases.

### 2.5. In Vitro Glucose-Lowering Activity

#### 2.5.1. Effects of Different Maturity and Resin Purification on Hypoglycemic Activity

Type 2 diabetes accounts for 90–95% of all diabetic patients, and its common pathogenesis is an unhealthy diet, which disrupts the balance between gastrointestinal carbohydrates and insulin secretion and action [57]. Inhibition of α-amylase and α-glucosidase is a prophylactic anti-diabetic treatment [58]. The hypoglycemic activity of proanthocyanidins is the result of multiple factors working together, which avoid blood glucose disorders through luminal effects, mainly including inhibiting carbohydrate digestion and absorption, stimulating intestinal hormone secretion, inhibiting endotoxin absorption, and improving gut microbiota [59]. Prolonged exposure of other organs to high glucose conditions may also lead to pathological changes, and proanthocyanidin intervention can effectively prevent and treat complications of type 2 diabetes. PCs from different sources have different structures, and even minor structural changes, including polymerization (mDP), C3 acylation, hydroxyl groups on the B-ring, and linkage types, can affect their blood glucose regulation [60]. Given the various mechanisms of PCs, different PCs with distinct structural characteristics remain to be explored.

**Figure 8:**
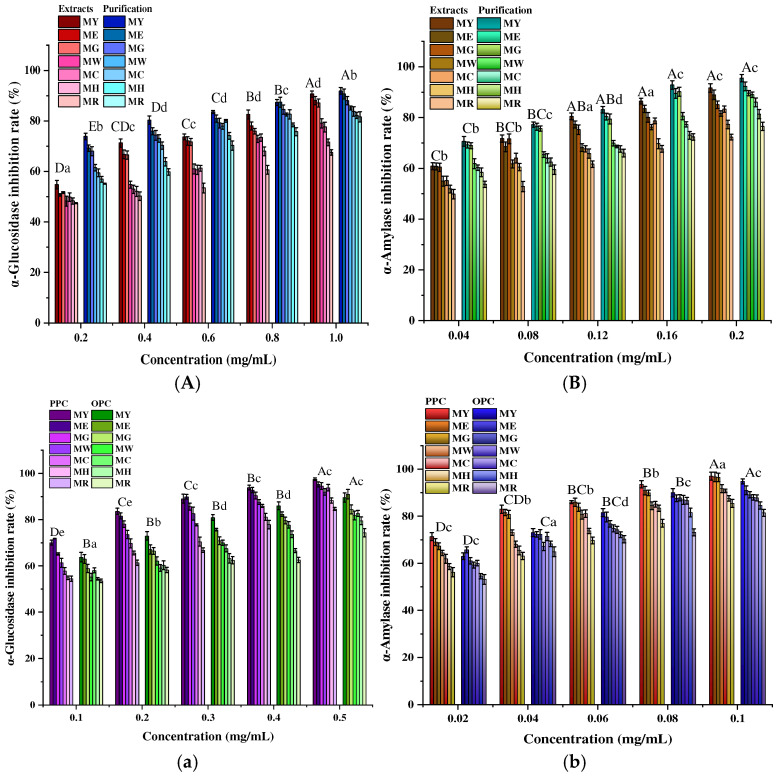
Inhibition of α-glucosidase (**A**) and α-amylase (**B**) by crude extract and purification; inhibition of α-glucosidase (**a**) and α-amylase (**b**) by PPC and OPC. MY, ME, MG, MW, MC, MH, and MR represent different periods of *Muzao*. Different letters indicate significant differences (*p* < 0.05). The values are expressed as means ± SD (*n* = 3). Uppercase letters indicate significant differences in antioxidant activity among different concentrations; lowercase letters indicate significant differences in antioxidant activity among different periods.

Figure 8A,B shows that proanthocyanidins exhibited a dose-dependent inhibition of α-glucosidase and α-amylase at concentrations of 0.1–0.5 mg/mL, with the purified product inhibiting these two enzymes (83%; 95.5%) more than the extract (67.5%; 72.3%), and with the decrease in concentration (0.5 mg/mL → 0.1 mg/mL) and the gradual maturation of *Muzao* (MY → MR), the inhibition rate decreased gradually. This may be due to the fact that the content of proanthocyanidins decreases during the ripening process of *Muzao*, which results in fewer substances with enzyme inhibitory activity, ultimately leading to a lower rate of inhibition of α-glucosidase and α-amylase. This is also consistent with the results of the changes in the content of some components of OPCs from *Muzao* at different times in the latter part of this paper. Huneif et al. [61] reported that the highest α-glucosidase and α-amylase activities in wild strawberry PCs were 71.69% ± 0.77% and 73.60% ± 1.63% for the crude extract and 87.63% ± 0.64% and 89.37% ± 0.54% for the purified extract, respectively. Meanwhile, Zhou et al. [41] isolated seven PC fractions from grape seeds, with the highest inhibition rates against these two enzymes reaching 95%. The α-amylase inhibition capabilities of PCs from loquat leaves and persimmon leaves were 86.54% and 83.81%, respectively [62], which are similar to the results of this study but higher than those of Pterocarpus erinaceus Poir (66.9% ± 2.0% and 53.2% ± 1.4%) [63]. Combined with the comparison of the antioxidant results of the purified products, it can be inferred that resin purification may have mitigated this adverse effect to some extent and improved the ability of proanthocyanidins to inhibit α-amylase and α-glucosidase. The results of this study indicate that MPC is a feasible alternative to pharmaceutical inhibitors for the treatment of diseases such as diabetes.

#### 2.5.2. Effect of Different Polymerization Degrees on Hypoglycemic Activity

In general, the α-amylase and α-glucosidase inhibitory activities of fruits were related to the composition and degree of polymerization of proanthocyanidins [64]. As can be seen from Figure 8a,b, the inhibition rates of α-glucosidase and α-amylase by PPC (84.6%; 85.5%) were slightly higher than those of OPC (74.3%; 81.4%), and there was no significant difference between the two (*p* > 0.05). Yamashita et al. [65] found that PCs with different degrees of polymerization promoted glucose uptake in L6 myotubes in a dose-dependent manner, with the high DP fraction showing greater inhibition of intestinal α-glucosidase activity than the low DP fraction. Jie et al. [66] found that proanthocyanidin extracts had a strong inhibitory effect on α-amylase and α-glucosidase, and the inhibitory effect was more pronounced with the increase in the level of polymerization and galactosyl or galacto-catechin units. Zhong et al. [67] observed that PCs extracted from grape seeds significantly inhibited α-amylase activity in the small intestine and pancreas of mice, especially with high polymers (1.95 and 1.73 times higher than oligomers). To some extent, this also proves our findings that there is a strong correlation between different degrees of polymerization and the hypoglycemic activity of MPC and that PPC has a higher inhibitory capacity than OPC.

### 2.6. FT-IR

**Figure 9:**
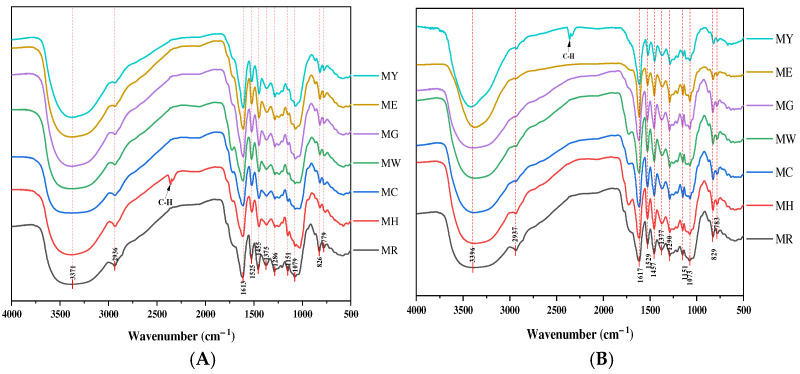
IR spectrum diagram of PPC (**A**) and OPC (**B**) in different periods of *Muzao*. MY, ME, MG, MW, MC, MH, and MR represent different periods of *Muzao*.

The chemical bonding and functional groups of the molecules of PPC and OPC from various periods of *Muzao* were investigated by FT-IR [68], and the infrared spectra are shown in Figure 9A,B. The spectral results showed that the proanthocyanidins of each period had similar infrared absorption groups in the scanning range. It indicates that the treatments of ultrasound and freeze-drying do not cause significant changes in the structure of proanthocyanidins, which is consistent with the results of some studies [69,70].

All samples have obvious absorption peaks (3397 cm^−1^) at 3600–3200 cm^−1^, which is caused by the tensile vibration of the hydroxyl structure of proanthocyanidin [71], and other peaks at 1000–900 cm^−1^ (825 cm^−1^), indicating that they are likely phenolic hydroxyl. The absorption peak at 1600–1650 cm^−1^ (1610 cm^−1^) and the characteristic absorption peak at 1600–1500 cm^−1^ (1520 cm^−1^) indicate that the three peaks at 1610, 1520, and 1460 cm^−1^ are the characteristic absorption peaks of the benzene ring [72].

Generally, a preliminary judgement can be made by the feature absorption of the infrared diagram of the chestnut shell extract with the original green sparrow or the original flower green as the main structural unit [73]. According to the quantitative change in hydroxyl groups on the B ring, polyprocanthocyanins are divided into structural units dominated by primary (the B ring contains three hydroxyl groups) or primary (the B ring contains two hydroxyl groups). The original has only one absorption peak at cm in the vibration frequency region (1540~1520 cm^−1^) and a strong absorption peak in the low-frequency fingerprint area (780~730 cm^−1^). As can be seen from the figure, four peaks (1375 cm^−1^, 1290 cm^−1^, 1150 cm^−1^, 1075 cm^−1^) at 1390~1000 are caused by C–O expansion vibration. The 825 cm^−1^ shows that 3 H is present on the benzene ring, and 777 cm^−1^ is a C–H plane structure. It can be inferred that the basic structural unit of proanthocyanidin isolated by *Muzao* purification is protoanthocyan.

### 2.7. SEM

**Figure 10:**
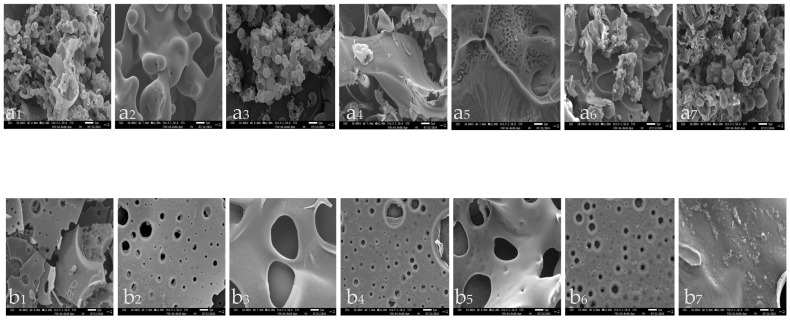
Electron microscopic scan of MPC: (**a_1–7_**) is 7 periods of OPC; (**b_1–7_**) is 7 periods of PPC. (**a**) refers to OPC; (**b**) refers to PPC; 1–7 refer to growth periods of *Muzao*: MY, ME, MG, MW, MC, MH, and MR.

Scanning electron microscopy (SEM) is one of the most effective tools to observe the differences in morphology and microstructure between samples [74]. Figure 10 shows the morphology of PPC and OPC at a magnification of 2.00 k. It can be observed that, at the same magnification, the OPC of all periods obtained under the optimal conditions showed a spherical associative structure and was not uniformly distributed. Whereas the surface of the PPC of all periods obtained by lyophilization was observed to be composed of interconnected sheets with porous slits on the surface. This is in agreement with the results of Rajakumari et al. [75].

The PPC periods do not differ very much, but b_3_ shows a typical three-dimensional mesh structure, which is similar to the results of Wang et al. [76]. The b_7_ period also has a lamellar structure, but the surface consists of spherical aggregates in clustered complexes. In contrast, OPC varies considerably from period to period. a_1–3_ have similar morphological features, with very distinct spherical structures, and the aggregation states are all in the form of clusters. a_4–5_ have similarities, with spherical structures adorning the inhomogeneous folds of the surface, with a_5_’s being a bit more pronounced. a_6–7_ are also more similar, with distinct aggregation states but with a shallower hierarchy of spherical structures compared to the previous three periods.

### 2.8. HPLC-M S/MS Analysis

To gain a clearer understanding of the composition and changes of OPCs during the development of *Muzao*, this study conducted a qualitative and quantitative analysis using HPLC-MS/MS. Mass spectrometry detected a total of 102 compounds, including 28 types of cyanidin derivatives, 16 types of peonidin derivatives, 15 types of pelargonidin derivatives, 14 types of delphinidin derivatives, 13 types of petunidin derivatives, 9 types of malvidin derivatives, 5 types of proanthocyanidins, and 2 types of flavonoids. More than 50 compounds were detected in each period, with little difference in the number of classes, but the specific compounds that were detected and their contents varied. Several previous studies [77,78,79] have illustrated and described similar types of phenolic compounds in dates.

**Figure 11:**
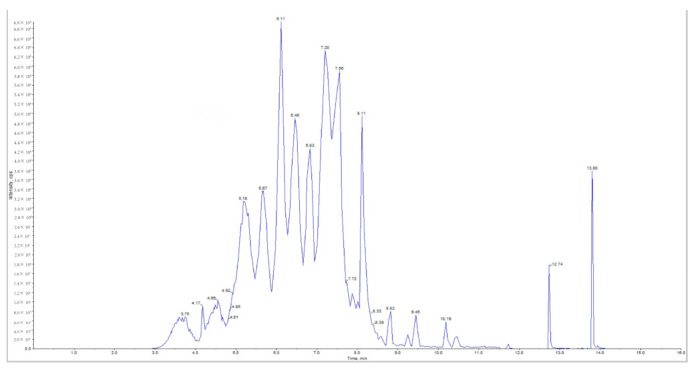
Total ion flow map (TIC).

According to the retention time, mass spectrometer, and standard characterization (Figure 11), the compounds contained in each period and their content are listed. It can be seen from Table S2 (Supplementary Materials) that the same compound varies in different periods. The obvious differences were delphinin-3-O-galactoside 0.79~6.89 μg/g and delphinin-3-O-glucoside 0.72~6.50 μg/g; centaurin-3-xylosy-galactoside 1.89~4.63 μg/g and centaurin-3-O-galactoside 1.63~12.72 μg/g; proanthocyanidin B1 1384.6~2178.9 μg/g, proanthocyanidin B2 94.27~316.19 μg/g, proanthocyanidin B3 1390.3~2169.7 μg/g, and proanthocyanidin B4 1424.2~2312.9 μg/g; quercetin 3-O-glucoside (isoquercetin) 98.92~330.12 μg/g. One of the possible reasons for the differences in the identified proanthocyanidin components is the concentration and purification procedure used [80]. Another possible reason is that the synthesis pathway of proanthocyanidins is activated by germination. Fan et al. [81] identified several important precursor substances related to pigments, and their contents increased significantly, indicating that red rice germination effectively stimulates the synthesis and accumulation of anthocyanins and proanthocyanidins.

Moreover, the trends of different compounds varied considerably during the development of *Muzao*. The contents of some substances were more and then less with the increase in development; for example, the contents of mallow pigment-3-O-glucoside, geraniol-3-O-rhamnopyranoside-5-O-glucoside, and proanthocyanidin B2 reached the highest in ME, the contents of sagittarius-malonyl-galonyl-glucoside-rhamnopyranoside reached the highest in MG, and the contents of proanthocyanidin B4 were the highest in MC and then gradually decreased. Xie et al. [82] found that proanthocyanidins in lychee fruits accumulated and then declined early in fruit development, but the time of peak proanthocyanidins was affected by the type of fruit and the variety. In the study of Wang et al. [83], it was also observed that there were some proanthocyanidins in the individual varieties of cranberries that reached a peak early in the development of the fruits and then gradually declined. Some substances, such as quercetin-3-O-glucoside (isoquercitrin), were found to gradually increase as the degree of development increased. Liu et al. [84] found that several quercetin classes such as quercetin-4′-O-glucuronide, quercetin-3-O-(2″-O-rhamnosyl)galactoside, and quercetin-3-O-neohesperidoside were all found to reach their highest values during the ripening stage from the secondary metabolites of the OPCs of the ginger development. There were also some substances whose contents gradually decreased with the continuous development of *Muzao*, such as cornflavin-3-xylosyl-galactoside, mallow pigment-3-O-glucoside, proanthocyanidin B1, and proanthocyanidin B3, which were highest in the first two periods and then gradually decreased. This trend was similar to the findings of Shi et al. [17]. These changes in OPC fractions during fruit development may be due to enzymatic activity and developmental regulation during fruit ripening [85].

## 3. Materials and Methods

### 3.1. Reagents and Instruments

*Muzao* was obtained from Shanxi Qikou Red Agricultural Technology Co., Ltd., Luliang City, China. The main reagents included proanthocyanidin standard ≥ 95%, Shanghai Yuanye Biotechnology Co., Ltd., Shanghai City, China; food-grade cellulase, Nanning Pangbo Bioengineering Co., Ltd., Nanning City, China; anhydrous ethanol, concentrated hydrochloric acid, ferric ammonium sulfate, methanol, n-butanol, DPPH, Tris-HCl buffer solution (pH 8.2), DNS, α-glucosidase, and α-amylase were all purchased from Shanghai Yuanye Technology Co., Shanghai City, China; salicylic acid, trichloroacetic acid, and ferric chloride were purchased from Tianjin Damao Chemical Reagent Factory, Tianjin City, China; ferrous sulfate and potassium ferricyanide were from Tianjin Windship Chemical Reagent Technology Co., Tianjin City, China.

The main equipment was CNC ultrasonic cleaner, Jiangsu Kunshan Ultrasonic Instrument Co., Ltd., Kunshan City, China; a pH meter, Shanghai Yidian Scientific Instrument Co., Ltd., Shanghai City, China; a low-speed centrifuge, Anhui Zhongke Zhongjia Scientific Instrument Co., Ltd., Hefei City, China; a full-wavelength enzyme labeling instrument, Thermo Fisher Scientific, Waltham, MA, USA; a rotary evaporator, Shanghai Yarong Biochemical Instrument Factory, Shanghai City, China; Fourier Transformer Infrared Spectroscopy (FT-IR), BRUKER TENSOR 27, Karlsruhe, Germany; a JSM-7500F Scanning Electron Microscope, JEOL, Tokyo, Japan; Ultra High-Performance Liquid Chromatography and Tandem Mass Spectrometry (LC-MS/MS), SCIEX, Plano, TX, USA.

### 3.2. Sample Pretreatment

**Figure 12:**
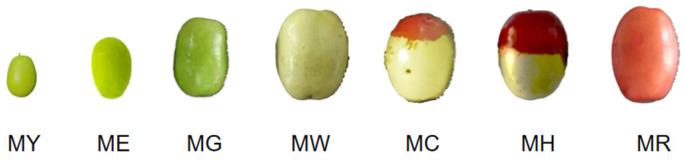
Different growth periods of *Muzao*: young fruit stage (MY), expansion stage (ME), green ripening stage (MG), white ripening stage (MW), cover red stage (MC), half red stage (MH), and full red stage (MR).

*Muzao*: According to the skin color and development degree in different periods, *Muzao* can be divided into 7 stages (Figure 12), including the young fruit stage (MY), expansion stage (ME), green ripening stage (MG), white ripening stage (MW), cover red stage (MC), half red stage (MH), and full red stage (MR). After picking, selecting, cleaning, pitting, crushing, and freeze-drying, *Muzao* is ground into powder by a high-speed crusher, and after passing through a 40-mesh sieve, the date powder is obtained, which is sealed with a self-sealing bag and kept in 4 °C environment for spare. The moisture content of dried jujube powder is 10.146 ± 0.130 g/100 g, and all experiments are based on dry matter.

Large hole resin: Macroporous resin: Referring to Li [86], slightly modified, prepare the appropriate amount of AB-8 macroporous resin; first soak with a 95% ethanol treatment for 24 h, and wash with water until there is no alcoholic odor, floaters, and white precipitates. Then, soak with 5% sodium hydroxide solution for 12 h; wash with water until neutral. Then, soak with 5% hydrochloric acid solution for 12 h, and wash with water until neutral. Finally, it is soaked in 95% ethanol and left for use.

### 3.3. Single-Factor and Response Surface Test

#### 3.3.1. Standard Curve Drawing of Proanthocyanidins

The proanthocyanidin content was determined by the HCL–n-butanol method [87]. The regression equation of the proanthocyanidin standard curve was obtained:

y = 0.0017x + 0.0449, R^2^ = 0.9997 (2)

This indicates that proanthocyanidins have a good linear relationship in the range of mass concentrations from 0 μg/mL to 200 μg/mL.

#### 3.3.2. Single Factor Test

Enzyme-assisted extraction is often combined with other technologies to obtain greater recovery of phenolic compounds and extract with more obvious biological activity [88]. Therefore, this study adopted an ultrasonic method combined with an enzymatic method to extract MPC. We accurately weighed 5 portions of *Muzao* powder 0.50 g in a centrifuge tube and examined the ultrasonic power (120, 140, 160, 180, 200 W), ethanol concentration (30, 40, 50, 60, 70%; *v*/*v*), material/liquid ratio (1:10, 1:20, 1:30, 1:40, 1:60; g/mL), temperature (40, 50, 60, 70, 80 °C), time (10, 20, 30, 40, 50 min), enzyme addition (0.25, 1.0, 1.5, 2, 3%; g/g), enzyme pH (3, 4, 5, 6, 7), and the effect of extracting twice on the yield of proanthocyanidins in *Muzao*, and we selected suitable one-factor conditions.

#### 3.3.3. Response Surface Experiment

The RSM is a scientific method for optimizing multi-factor experiments. According to the results of the single-factor experiment, the factors that have a significant influence on the response value were selected. The main factors selected were temperature (°C), solid/liquid ratio (g/mL), enzyme addition amount (%), and MPC yield (%), which was taken as the response value. A series of experiments were designed using the response surface method (RSM) using the design-expert 13 software to optimize the extraction conditions for the three levels of these three factors. The ANOVA analysis of variance was used to test the significance of the regression model to confirm that the model had a better fitting degree and prediction ability.

To verify the predictive accuracy of the regression model, it is usually necessary to repeat the experiment under optimal conditions and compare the predicted values of the regression model with the actual values. If there is no significant difference between the model and the validation results (*p* > 0.05), it indicates that the conditions optimized by the RSM can be applied to the extraction of MPC.

### 3.4. Optimization Tests for MPC Purification by Macroporous Resins

#### 3.4.1. Static Adsorption and Desorption Experiments

Weigh five portions of 0.5 g AB-8 resin and adjust the sample concentration (1, 2, 3, 4, 5 mg/mL), sample pH value (1.0, 3.0, 5.0, 7.0, 9.0), desorption solution concentration (10%, 30%, 50%, 70%, 90%), and desorption solution pH value (2.0, 4.0, 6.0, 8.0, 10.0) and shake for treatment. Measure the concentration of filtrate samples at regular intervals, calculate the adsorption and desorption rates of AB-8 resin on *Muzao* proanthocyanins according to Equations (3) and (4), and investigate the adsorption and desorption effects of various factors.

(3) $$\mathrm{Adsorption}\;\mathrm{rate}=\frac{C_{0}-C_{1}}{C_{0}}\times 100\%$$

(4) $$\mathrm{Desorption}\;\mathrm{rate}=\frac{C_{2}}{C_{0}-C_{1}}\times 100\%$$

where *C*_0_ is the MPC concentration of the adsorption sample; *C*_1_ is the MPC concentration of the adsorption filter; *C*_2_ is the MPC concentration of the desorption collection solution.

#### 3.4.2. Dynamic Adsorption and Desorption Experiments

Based on the static experiments, the wet column was used to investigate the effects of sample volume (0.5~20 bv), sample flow rate (0.5, 0.75, 1, 1.25, 1.5 mL/min), eluent pH (2.0, 4.0, 6.0, 8.0, 10.0), eluent concentration (30%, 40%, 50%, 60%, 70%), and eluent dosage (0.25~4 bv) on resin adsorption and desorption. The effluent was collected in 0.5 bv (1 bv = 30 mL), the concentration of date proanthocyanidins in the effluent was determined, and the leakage curves were plotted to investigate the effects of various factors on the adsorption and desorption of the resin.

### 3.5. In Vitro Antioxidant Capacity

#### 3.5.1. DPPH Free Radical Scavenging Ability

Referring to the method of Shi et al. [44], the sample to be measured was dissolved into a mixed homogeneous solution, diluted into the required gradient mass concentration, and left for 30 min away from light to determine the absorbance value at 517 nm. The DPPH radical clearance rate of the sample to be measured was calculated according to Equation (5).

(5) $$\mathrm{Clearance}\;\mathrm{rate}=\frac{A_{C}-(A_{i}-A_{j})}{A_{C}}\times 100\%$$

where *A_i_* is the absorption value of DPPH and blank solvent mixture; *A_j_* is the absorption value of DPPH and sample; *A_c_* is the light absorption value of sample mixture and blank solvent.

#### 3.5.2. Resonance of Hydroxyl Radical by Salicylic Acid

After the FeSO_4_ solution, salicylic acid solution, distilled water, and H_2_O_2_ solution were added to the PCR plate, a 37 °C water bath was heated for 15 min, and the absorbance was measured at 510 nm. This was repeated 3 times to calculate the clearance rate according to Equation (5).

#### 3.5.3. Reducing Force Determination

According to Zhang [89], the reducing power was measured by adding 0.1 mL of the sample to the test tube, along with 0.5 mL of phosphate buffer solution (0.2 mol/L, pH 6.6) and 0.5 mL of 10% trichloroacetic acid. The sample was left to stand at room temperature for 10 min. Then, we took 0.5 mL of reaction solution and added 0.5 mL of distilled water and 0.1 mL of 0.1% ferric chloride solution. The absorbance at 700 nm after 10 min of reaction was measured.

#### 3.5.4. Superoxide Anion

Referring to the method of Peng et al. [45] with slight modifications, take 4.5 mL of 0.05 mL/L Tris-HCl buffer solution (pH 8.2) and mix it with 1 mL of ultrapure water. Incubate at 25 °C for 20 min in a water bath. Then, quickly add 1 mL of sample solution and 5 mL of 25 mol/L solution of pyrogallol and place them in a 25 °C water bath for 5 min. Finally, add 1 mL of 8 mol/L hydrochloric acid to end the reaction. Measure the absorbance value at 320 nm and perform parallel detection three times.

### 3.6. Glucose-Lowering Activity

#### 3.6.1. Determination of the Ability to Inhibit α-Glucosidase Activity

Following the method of Huneif et al. [61], 0.4 mL of pH 6.8 phosphate buffer solution, 0.2 mL of sample solution, and 0.2 mL of α-glucosidase solution were sequentially added to a test tube. After mixing, they were immersed in a water bath at 37 °C for 15 min. Then, we added 0.4 mL of 2.5 mmol/L pNPG solution, continued the reaction for 15 min, and finally, added 5 mL of 0.1 mol/L sodium carbonate solution to terminate the reaction. We measured its absorbance at 405 nm. The formula for calculating the inhibition rate is as follows:

(6) $$\alpha \text{-}\mathrm{glucosidase}\;\mathrm{inhibition}\;\mathrm{rate}=\frac{A_{0}-(A_{1}-A_{2})}{A_{0}}\times 100\%$$

where *A*_1_ is the sample group; *A*_2_ is the color interference group; *A*_0_ is the blank group.

#### 3.6.2. Determination of the Ability to Inhibit α-Amylase Activity

Add 0.5 mL of α-amylase solution and 0.5 mL of the sample to be tested to the tube at 37 °C for 30 min; add 1 mL of 1% starch solution for 15 min; add 1 mL DNS of color developer; use boiling water bath for 5 min and then immediately cool to room temperature in the ice bath to 25 mL, mix, and measure the absorbance at 540 nm. The inhibition rate is calculated as follows:

(7) $$\alpha \text{-}\mathrm{amylase}\;\mathrm{inhibition}\;\mathrm{rate}=\frac{B_{0}-(B_{1}-B_{2})}{B_{0}}\times 100\%$$

where *B*_1_ is the sample group; *B*_2_ is the color interference group; *B*_0_ is the blank group.

### 3.7. Structural Characterization

#### 3.7.1. Fourier Infrared Spectrum Detection (FT-IR)

The proanthocyanidin sample and KBr were fully ground and pressed at a ratio of 1:100 for FT-IR testing, with a wave range of 4000 to 400 cm^−1^, 64 scans, and a resolution of 4 cm^−1^.

#### 3.7.2. Electron Microscope Scanning (SEM)

Place the OPC and PPC freeze-dried samples prepared at different stages on the sample stage and perform gold spraying treatment. Observe the microstructure of PPC and OPC using a scanning electron microscope at an acceleration voltage of 10 kV and a magnification of 2.0 k×.

#### 3.7.3. UPLC-Q-TOF-MS Identified the OPC Components

Quid phase conditions mainly include the following: column: ACQUITY BEH C18 1.7 µm, 2.1 mm × 100 mm; mobile phase: Phase A is ultrapure water (adding 0.5% formic acid), phase B is methanol (adding 0.5% formic acid); elution gradient: 0.00 min B phase: 5%, 6.00 min to 50%, 12.00 min; flow rate 0.35 mL/min; column temperature 40 °C; sample volume 2 μL.

The MS conditions mainly include the following: electrospray ion source (Electrospray Ionization, ESI) temperature 550 °C, MS voltage 5500 V in positive ion mode, air curtain gas (Curtain Gas, CUR) 35 psi. In Q-Trap 6500+, each ion pair is scanned according to the optimized decluster voltage (Declustering Potential, DP) and collision energy (Collision Energy, CE).

### 3.8. Data Processing and Statistical Analysis

Each experiment was set up with three replicates, and data were organized using Excel 2010. According to the one-way ANOVA test analysis in the general linear model of SPSS 27, the significance level was *p* < 0.05, and the results were expressed as mean ± standard deviation. The chart was plotted using Origin 2022.

## 4. Conclusions

This experiment conducted a single-factor study using ultrasonic-assisted enzymatic extraction. Through response surface methodology and validation experiments, the optimal conditions for ultrasonic-enzymatic extraction of MPCs were determined as follows: an ethanol concentration of 50%, solid-to-liquid ratio of 1:41, ultrasonic time of 30 min, ultrasonic power of 200 W, enzymatic hydrolysis temperature of 64 °C, enzyme addition of 1.14%, and enzymatic hydrolysis pH of 5. Under these conditions, the yield of MPCs was 2.01%. On this basis, the optimal conditions for the purification of the extract with AB-8 macroporous resin were the following: a sample concentration of 2 mg/mL, sample pH of 5, sample volume of 1.6 bv, sample flow rate of 1 mL/min, elution volume of 3 bv, ethanol concentration of 70%, eluent pH of 4, and elution volume of 2.5 bv. The content of MPC was increased by nearly 11-fold after the purification, and its antioxidant and hypoglycemic activities were also significantly enhanced. Then, the purified extract was separated into two fractions, PPCs and OPCs, using ethyl acetate extraction. Our biological activity tests revealed that OPCs demonstrated notably stronger antioxidant activity than PPCs, whereas PPCs showed a slightly greater inhibitory effect on α-glucosidase activity compared to OPCs. The infrared spectra indicated that neither the enzymatic nor the ultrasonic extraction methods altered the basic structure of the proanthocyanidins. SEM images showed significant differences in the morphological characteristics of PPCs and OPCs from proanthocyanidins at different growth stages, with PPCs mostly exhibiting a polymerized, smooth, and porous structure, and OPCs consistently displaying a spherical and connected structure. Mass spectrometry analysis detected a total of 102 compounds in OPC, including 28 cyanidin-type compounds, 5 proanthocyanidin-type compounds, 2 flavonoid-type compounds, etc., among which the content of proanthocyanidin B3 was ≥1390.3 μg/g. In this study, all the analytical techniques identified that MY is a potential source of OPCs, which can not only be used in the production of daily foods, such as juice, bread, and breakfast powder, but also can be used to make health care products such as chewable tablets, capsules, and oral liquids, and they can also be added to facial masks and cosmetics to enhance their efficacy, which has a wide range of industrial application value. This result provides theoretical support for exploring a new source of PCs, developing functional foods, and enhancing the added value of *Muzao*.

## Figures and Tables

**Table 1:** Design of response surface and test results.

Run Order	Level of Factors			MPC Yield %
	A (Temperature, °C)	B (Liquid to Material Ratio)	C (Enzyme Addition, %)	
1	60	30	0.5	1.79 ± 0.08
2	60	40	1	2.04 ± 0.05
3	60	30	1.5	1.45 ± 0.04
4	60	40	1	2.00 ± 0.02
5	60	40	1	1.97 ± 0.07
6	60	50	1.5	1.84 ± 0.05
7	70	40	1.5	1.99 ± 0.05
8	60	40	1	2.10 ± 0.05
9	70	30	1	1.60 ± 0.03
10	50	50	1	1.35 ± 0.04
11	60	50	0.5	1.59 ± 0.04
12	50	40	0.5	1.50 ± 0.02
13	70	40	0.5	1.87 ± 0.06
14	50	30	1	1.56 ± 0.04
15	70	50	1	1.66 ± 0.07
16	60	40	1	1.91 ± 0.04
17	50	40	1.5	1.61 ± 0.03

## Data Availability

The original contributions presented in the study are included in the article, and further inquiries can be directed to the corresponding author.

## Supplementary Materials

The following supporting information can be downloaded at: https://www.mdpi.com/article/10.3390/molecules30030619/s1, Table S1: The ANOVA table for the regression equation; Table S2: Content of components of OPC at different periods of *Muzao*.
