# Supplementary material for: Optimization of Ultrasonic-Enzyme Synergistic Extraction of Proanthocyanidins from Jujube: Purification, Characterization, and Bioactivity Study
Source: Molecules. 2025 Jan 31;30(3):619. doi: 10.3390/molecules30030619 (PMC11820555; doi:10.3390/molecules30030619)
Supplement: Supplementary file 1 [file molecules-30-00619-s001.zip › Table S1. The ANOVA table for the regression equation.pdf]

Table S1. The ANOVA table for the regression equation

Source	Sum of Squares	df	Mean Squares	F-value	p-value	Significance
Model	0.7811	9	0.0868	8.96	0.0043	**
A	0.1513	1	0.1513	15.12	0.0055	**
B	0.0002	1	0.0002	0.0207	0.8898	
C	0.0025	1	0.0025	0.2531	0.6304	
AB	0.0182	1	0.0182	1.88	0.2124	
AC	0.0000	1	0.0000	0.0026	0.9609	
BC	0.0870	1	0.0870	8.99	0.0200	*
A2	0.1572	1	0.1572	16.24	0.0050	**
B2	0.3030	1	0.3030	31.30	0.0008	***
C2	0.0196	1	0.0196	2.03	0.1977	
Residual	0.0678	7	0.0097			
Lack of Fit	0.0472	3	0.0157	3.07	0.1534	Not significant
Pure Error	0.0205	4	0.0051			
Cor Total	0.8488	16				

Note: *** means extremely significant ($p < 0.001$); * * means Very significant ($p < 0.01$); * means significant ($p < 0.05$).
