# Supplementary material for: Optimization of Ultrasonic-Enzyme Synergistic Extraction of Proanthocyanidins from Jujube: Purification, Characterization, and Bioactivity Study
Source: Molecules. 2025 Jan 31;30(3):619. doi: 10.3390/molecules30030619 (PMC11820555; doi:10.3390/molecules30030619)
Supplement: Supplementary file 1 [file molecules-30-00619-s001.zip › Table S2. Content of components of OPC at different periods of Muzao.pdf]

Table S2. Content of components of OPC at different periods of *Muzao*

NO.	Compounds	RT(min)	Formula	Stage-Content（ug/g）						
				MY	ME	MG	MW	MC	MH	MR
1	Delphinidin-3-O-galactoside	5.26	C21H21O	4.37±	1.25±	4.83±	0.79±	0.83±0	4.55±	6.89±
2	Delphinidin-3-O-glucoside	5.27	C21H21O [illegible]	4.06± [illegible]	1.32± [illegible]	4.45± [illegible]	0.72± [illegible]	0.93±0 [illegible]	4.22± [illegible]	6.50± [illegible]
3	Delphinidin-3-O-(6''-O-tartaryl)glucoside	8.31	C25H25O [illegible]	1.93± [illegible]	1.62± [illegible]	2.03± [illegible]	1.55± [illegible]	1.27±0 [illegible]	1.26± [illegible]	1.98± [illegible]
4	Delphinidin-3-O-(6''-O-coumaroyl)rhamnoside-5-O-	9.21	C36H37O	0.24±	0.67±	0.53±	0.25±	0.48±0	0.48±	0.72±
5	Delphinidin-3-O-rutinoside-5-O-glucoside	5.89	C42H47O [illegible]	0.59± [illegible]	0.41± [illegible]	1.65± [illegible]	0.21± [illegible]	0.21±0 [illegible]	2.19± [illegible]	2.26± [illegible]
6	Delphinidin-3-O-sophoroside	4.81	C27H31O	0.24±	0.08±	0.53±	0.07±	0.13±0	0.57±	0.77±
7	Delphinidin-3-O-rutinoside	5.64	C27H31C [illegible]	1.53± [illegible]	0.58± [illegible]	1.98± [illegible]	0.26± [illegible]	0.34±0 [illegible]	1.47± [illegible]	1.28± [illegible]
8	Delphinidin-3-O-(coumaroyl)glucoside-5-O-galactoside	5.90	C36H37O	0.26±	0.28±	1.05±	0.12±	0.12±0	0.69±	0.76±
9	Delphinidin-3-O-(6''-O-acetyl)galactoside	10.05	C23H23O	0.11±	0.08±	0.07±	0.11±	0.067±	0.08±	0.10±
1	Cyanidin-3-xylosyl-galactoside	5.84	C26H29O	4.63±	4.56±	4.33±	4.15±	3.79±0	3.18±	1.89±
2	Cyanidin-3-O-glucoside-5-O-galactoside	3.25	C27H31O 16+	0.005 ±0.00	0.006 ±0.00	0.003 ±0.00	0.006 ±0.00	0.003± 0.0002	0.005 ±0.00	0.003 ±0.00
3	Cyanidin-3-O-sophoroside	5.68	C27H31C	1.46±	0.32±	1.30±	0.18±	0.24±0	0.73±	0.90±
4	Cyanidin-3-O-(tartaryl)rhamnoside-5-O-glucoside	6.30	C31H35O	0.15±	0.13±	1.62±	0.14±	0.10±0	0.17±	0.09±
5	Cyanidin-3-O-arabinoside	6.45	C20H19C lO10	0.07± 0.003	0.06± 0.002	0.07± 0.002	0.08± 0.001	0.04±0 .002	0.10± 0.003	0.06± 0.001

NO.	Compounds	RT(min)	Formula	Stage-Content（ug/g）							Class
				MY	ME	MG	MW	MC	MH	MR	
6	Cyanidin-3-O-galactoside	5.72	C21H21O11+	6.56±0.01	4.08±0.02	12.72±0.05	1.63±0.03	1.89±0.[illegible]	6.55±0[illegible]	10.36±0.[illegible]	
7	Cyanidin-malonyl-malonyl-glucoside-	10.02	C33H35O	0.01±0.00	0.02±0.	0.03±0	0.01±0	0.01±0.	0.02±0	0.01±0.0	
1	Procyanidin B1	4.19	C30H26O12	2057.7±2.24	2178.9±1.15	2026.5±0.30	2098.5±0.47	2016.5±0.32	1671.6±0.43	1384.6±0.30	
[illegible]	Procyanidin B2	[illegible]	C36H37O[illegible]	94.27±0.0[illegible]	316.19[illegible]	304.86[illegible]	250.22	299.63±0.04	261.08±0.20	142.3±0.01	Delphinidin
3	Procyanidin B3	4.18	C30H26O	2044.1±1.89	2169.7±0.35	2134.1±0.60	2124.2±0.18	2144.2±0.05	1741±0.33	1390±0.01	
4	Procyanidin B4	5.13	C30H26O12	1677.7±0.28	1904.6±0.41	2129.6±0.31	2085.6±0.44	2312.9±0.09	1889.4±0.25	1424.2±0.07	
1	Malvidin-3-O-galactoside	7.39	C23H25C	0.06±0.00	0.09±0.	0.05±0	0.08±0	0.07±0.	0.04±0	0.05±0.0	
2	Malvidin-3-O-(6''-O-feruloyl)galactoside	12.74	C33H33O15+	0.01±0.001	0.007±	0.007±0.0001	0.01±0002	0.008±0.001	0.008±0.0002	0.01±0.001	
3	Malvidin-3-O-glucoside	7.39	C23H25O	0.09±0.00	0.12±0.	0.09±0	0.09±0	0.09±0.	0.06±0	0.05±0.0	
1	Peonidin-3-O-araboside-glucoside	6.70	C27H31O	1.79±0.02	2.07±0.	1.14±0	3.56±0	1.05±0.	2.74±0	2.82±0.0	
2	Peonidin-3,5-O-diglucoside	5.53	C28H33O	0.03±0.00	0.02±0.	0.01±0	0.03±0	0.03±0.	0.03±0	0.04±0.0	Cyanidin
3	Peonidin-3-O-glucoside	7.22	C22H23O	0.35±0.03	0.15±0.	0.42±0	0.12±0	0.10±0.	0.25±0	0.38±0.0	
1	Petunidin-caffeoyl-rhamnoside-glucoside	7.08	C37H39O	0.02±0.00	0.01±0.	0.03±0	0.01±0	0.01±0.	0.03±0	0.02±0.0	
2	Petunidin-glucoside-galactoside	7.06	C28H33O17+	0.07±0.001	0.07±0.002	0.23±0.01	0.02±0.001	0.04±0.002	0.14±0.001	0.14±0.02	

NO.	Compounds	RT(min)	Formula	Stage-Content（ug/g）							Class
				MY	ME	MG	MW	MC	MH	MR	
3	Petunidin-3-O-(6-O-p-coumaroyl)-glucoside	10.69	C31H29O14+	0.96±0.01	2.86±0.04	2.24±0.11	0.78±0.01	0.74±0.01	2.14±0.01	1.72±0.005	Cyanidin
1	Naringenin	12.74	C15H12O5	0.47±0	1.11±0	0.67±0.	0.39±0	0.65±0.	0.46±0	0.28±0	
2	Quercetin-3-O-glucoside	9.45	C21H20O12	330.12±1.10	253.17±0.88	215.35±0.21	98.925±0.02	152.21±0.05	148.64±0.01	166.72±0.02	
[illegible]	Pelargonidin-3-O-(6''-O-malonyl)galactoside	[illegible]	C24H23O13+	0.85±0.01	1.76±0.03	1.08±0.01	0.04±0.002	1.33±0.01	1.08±0.03	0.50±0.02	Procyanidin
2	Pelargonidin-3-O-rhamnoside-5-O-glucoside	4.09	C27H31O14+	161.12±0.10	184.19±0.05	171.36±0.01	162.64±0.01	155.92±0.01	124.81±0.01	96.54±0.01	
											Malvidin
											Peonidin
											Petunidin

**Class**
Petunidi
flavonoid
Pelargonidin
